# Ankle‐brachial index, arterial stiffness, and biomarkers in the prediction of mortality and outcomes in patients with end‐stage kidney disease

**DOI:** 10.1002/clc.23188

**Published:** 2019-04-30

**Authors:** Kenichiro Otsuka, Koki Nakanishi, Kenei Shimada, Haruo Nakamura, Hitoshi Inanami, Hiroki Nishioka, Kohei Fujimoto, Noriaki Kasayuki, Minoru Yoshiyama

**Affiliations:** ^1^ Department of Cardiovascular Medicine Ishikiri‐seiki Hospital Higashi‐osaka Japan; ^2^ Department of Cardiovascular Medicine Baba Memorial Hospital Sakai Japan; ^3^ Department of Cardiovascular Medicine Kashiba‐seiki Hospital Kashiba Japan; ^4^ Department of Cardiovascular Medicine Osaka City University Graduate School of Medicine Osaka Japan

**Keywords:** arterial stiffness, biomarker, dialysis, peripheral arterial disease, prognosis

## Abstract

**Background:**

Although ankle‐brachial index (ABI) and brachial‐ankle pulse wave velocity (baPWV) are significant predictors of major adverse cardiovascular event (MACE), their prognostic value in association with biomarkers has not been fully evaluated in patients with end‐stage kidney disease (ESKD).

**Hypothesis:**

We hypothesized that ABI/baPWV would provide better prognostic value independent of biomarkers in ESKD patients.

**Methods:**

This study included 104 ESKD patients treated with maintenance hemodialysis who underwent ABI and baPWV examinations and laboratory tests, including brain‐natriuretic peptide, high‐sensitive cardiac troponin T (hs‐cTnT), and high‐sensitive C‐reactive protein (hs‐CRP). MACE was defined as a composite event of all‐cause death, acute coronary syndrome, and stroke.

**Results:**

During a mean follow‐up of 3.6 ± 1.7 years, a total of 51 MACE were observed. The independent factors associated with MACE were age >75 years (adjusted hazard ratio [HR], 2.15; *P* < .05), abnormal ABI (adjusted HR, 2.01; *P* < .05), left ventricular ejection fraction (LVEF) <50% (adjusted HR, 3.33; *P* < .001), the upper tertile of hs‐cTnT (adjusted HR, 2.77; *P* < .05), and hs‐CRP (HR, 1.96; *P* < .05). However, baPWV did not remain as an independent predictor of MACE in the entire cohort and also in patients without abnormal ABI. The combination of predictors improves the predictive value of MACE, providing increased HR with 4.00 for abnormal ABI + hs‐CRP, 4.42 for abnormal ABI + hs‐cTnT, and 7.04 for abnormal ABI + LVEF <50% (all *P* < .001).

**Conclusion:**

Abnormal ABI is a robust predictor of MACE independent of biomarkers and their combination provides better risk stratification compared with a single predictor in ESKD patients.

## 1 INTRODUCTION

End‐stage kidney disease (ESKD) is characterized by multiple risk factors which pose increased risks of mortality and major adverse cardiovascular events (MACE).^1^, ^2^ The mechanisms underlying the association between ESKD and MACE include highly prevalent comorbidities such as coronary artery disease, cerebrovascular disease, and peripheral artery disease (PAD).^2^, ^3^, ^4^ Accumulating evidence suggests that arterial stiffness and ankle‐brachial index (ABI) are well correlated with atherosclerotic burden and are strong predictors of MACE.^5^, ^6^, ^7^, ^8^ Among the various indices of arterial stiffness, brachial‐ankle (ba) pulse wave velocity (PWV) is an established tool for the noninvasive assessment of arterial stiffness in clinical practice.^9^, ^10^ However, measurements of baPWV can be influenced by the presence of PAD and hemodynamic condition after hemodialysis (HD) session.^11^, ^12^, ^13^ Therefore, the prognostic value of baPWV in ESKD patients is still discussed.^14^

With the widespread clinical application of standardized assays, several biomarkers and multiple biomarker approach enable the accurate prediction of the long‐term prognosis of ESKD patients treated with HD.^15^, ^16^, ^17^ Previous studies have demonstrated that cardiac troponin (cTn), brain‐natriuretic peptide (BNP), and C‐reactive protein (CRP) are the robust predictors of MACE independent of traditional risk factors and two‐dimensional thoracic Doppler echocardiography (TTDE) findings.^18^, ^19^, ^20^, ^21^ However, the prognostic significance of ABI/PWV in association with biomarkers have not been fully investigated in ESKD patients. In addition, the heterogeneous risks of ESKD patients might require risk assessment approach from multiple aspects. The aim of this study was to investigate the prognostic value of ABI/PWV in association with biomarkers and to examine whether biomarkers would provide additional predictive value on ABI/PWV in ESKD patients treated with HD.

## 2 METHODS

### 2.1 Study population

During the period between April 2012 and February 2013, a total of 104 consecutive ESKD patients treated with HD for more than 1 year at the Ishikiri‐seiki Hospital were enrolled in this study. The exclusion criteria were as follows: (a) patients who were hospitalized within the past 1 month due to acute coronary syndrome (ACS) or heart failure; (b) patients with systemic infection or chronic inflammatory diseases; and (c) patients with symptomatic severe valvular heart diseases requiring surgical intervention. The present study was approved by the Ethics Committee of Ishikiri‐seiki Hospital and was conducted in accordance with the institutional regulations and the Declaration of Helsinki. All the study patients gave written informed consent for the enrollment in this study.

### 2.2 Laboratory data collection

All the patients were required to provide baseline plasma samples before HD session on the day underwent HD session, including hemoglobin, hemoglobin A1c, cholesterol profiles, BNP, high‐sensitive cTnT (hs‐cTnT), and high‐sensitive CRP (hs‐CRP). The samples were centrifuged within 60 minutes and stored at −80°C for further analysis. hs‐cTnT was measured by an electro chemiluminescence immunoassay (ECLIA) with the EClusys hs‐cTnT Roche diagnostic assay. In the hs‐cTnT assay, the 99th percentile of the upper reference limit and the lower limit of detection were 0.014 and 0.003 ng/mL, respectively. BNP was measured with a specific immunoradiometric assay for human BNP (ARCHITECT BNP‐JP; ABBOTT JAPAN Co, Ltd, Tokyo, Japan). The inter‐ and total coefficient variation for BNP was 1.1% to 5.1% and 2.3% to 5.3%, respectively. Hs‐CRP was measured using N‐latex CRP II (Dade Behring Inc., Marburg, Germany) with a coefficient variation of 3.1% at 0.5 mg/L.

### 2.3 ABI/PWV and TTDE examinations

ABI/PWV and TTDE examinations were performed in all study patients in our laboratory at appropriate temperature according to the guideline.^6^, ^22^ ABI/PWV were measured at the same day on the next day of HD session. After a 10‐minute rest, baPWV was measured with a Colin Waveform Analyzer (form PWV/ABI; Colin Medical Technology, Komai, Japan) in the spine position. baPWV was measured by an automatic oscillometric method. The automatic device measures the time delay between the rapid upstroke of the feet of simultaneously recorded pulse waves in posterior tibial arteries and the brachial artery in the nonblood access side. The distance between the measurement sites in the brachial and ankle was measured with a tape over the surface of the body. ABI was calculated as the ratio of the systolic blood pressure measured at the ankle to that at the brachial artery, with no blood access. The lower value of ankle blood pressure was used for the assessment of ABI. Abnormal ABI was defined as <0.90 or >1.40.^6^ TTDE examination was also performed after the HD session at the study enrollment using a commercially available system (the Vivid 7; General Electric, Milwaukee, Wisconsin) by trained cardiac echo sonographers. Left ventricular ejection fraction (LVEF) was calculated by using Simpson method from apical 4‐ and 2‐chamber views, as previously reported.^21^ In the present study, LVEF <50% was defined as decreased LVEF.

### 2.4 Outcome assessment

The prognostic status of all patients was assessed by reviewing the medical records of the Ishikiri‐seiki Hospital. The primary endpoint was MACE consists of all‐cause death, ACS requiring coronary revascularization, and stroke. The occurrence of MACE was judged by two cardiologists (N.H. and K.N.) who were blinded to the results for patient characteristics and measurements such as ABI/PWV, TTDE, and biomarkers.

### 2.5 Statistical methodology

All statistical analyses were performed with the SPSS 22.0 (SPSS Japan Inc., Tokyo, Japan). Categorical variables were summarized as frequencies with percentages. Continuous variables were presented as mean ± SD or median (interquartile range, IQR) appropriately. The measurements of baPWV and biomarkers including BNP, hs‐cTnT, and hs‐CRP were divided into tertiles for the entire group (n = 104) and the normal ABI group (0.9 to 1.4; n = 73), respectively. The upper tertile of outcome measures was compared to the measurements of the middle and the lower tertile in the univariate and multivariate Cox hazard model. Multivariate analysis using Cox proportional hazard model was carried out to determine the independent predictors of MACE, which included the predictors of MACE as shown in univariate analysis at a level of *P* value <.1. Hazard ratios (HRs) for MACE of the combination of predictors were estimated with the use of an unadjusted Cox proportional‐hazards model. The cumulative rates for MACE were evaluated using Kaplan‐Meier analysis with log‐rank test for combination of the presence or absence of the predictors. A two‐sided *P* value <.05 was considered statistically significant.

## 3 RESULTS

### 3.1 Patient characteristics and outcomes

Baseline patient characteristics are presented in Table 1. The mean age of the entire group was 71 ± 7 years, 63% were male (n = 66), and 31 (30%) patients had abnormal ABI (<0.9 and/or >1.4). In the entire group, the median value of baPWV was 2013 (IQR, 1775‐2632) cm/second. Decreased LVEF (<50%) was observed in 19% of the study patients. The median value of the biomarkers including BNP, hs‐cTnT, and hs‐CRP, were 368.8 (IQR, 164.2‐749.8) pg/mL, 0.063 (IQR, 0.042‐0.095) ng/mL, and 0.08 (IQR, 0.032‐0.258) mg/dL, respectively.

**Table 1:** Patient characteristics

Number of patients, the entire group	n = 104
Age, years	71 ± 7
Male, n (%)	66 (63)
Body mass index, kg/m^2^	21.7 ± 3.5
Hypertension, n (%)	74 (71)
Diabetes mellitus, n (%)	39 (38)
Dyslipidemia, n (%)	47 (45)
Prior myocardial infarction, n (%)	10 (10)
Prior coronary revascularization, n (%)	29 (28)
Prior stroke, n (%)	15 (14)
Smoking history, n (%)	35 (34)
Systolic blood pressure, mm Hg	138 ± 22
Heart rate, bpm	75 ± 12
LVEF <50%, n (%)	20 (19)
baPWV, cm/s	2262 ± 748
Abnormal ABI, n (%)	31 (30)
Medication	
ACE‐I/ARB, n (%)	42 (40)
Calcium channel blocker, n (%)	52 (50)
Beta blocker, n (%)	29 (28)
Statin, n (%)	14 (13)
Aspirin, n (%)	48 (46)
Laboratory test	
Hemoglobin A1c, %	5.5 ± 0.9
Hemoglobin, g/dL	10.8 ± 1.1
HDL‐cholesterol, mg/dL	49 ± 12
LDL‐cholesterol, mg/dL	87 ± 29
BNP, pg/mL	368.8 (164.2‐749.8)
hs‐TnT, ng/mL	0.063 (0.042‐0.095)
hs‐CRP, mg/dL	0.08 (0.032‐0.258)

*Note*: Variables are presented as number (%), mean ± SD, or median (IQR). The outcome measures of baPWV and biomarkers including BNP, hs‐cTnT, and hs‐CRP were divided into tertiles of the entire group (n = 104).

Abbreviations: ABI, ankle‐brachial index; ACE‐I, angiotensin‐converting enzyme inhibitor; ARB, angiotensin II receptor blocker; ba‐PWV, brachial‐ankle‐pulse wave velocity; BNP, brain‐natriuretic protein; HDL, high‐density lipoprotein; hs‐CRP, high‐sensitive C‐reactive protein; hs‐cTnT, high‐sensitive cardiac troponin T; hs‐TnT, high‐sensitive troponin T; IQR, interquartile range; LDL, low‐density lipoprotein; LVEF, left ventricular ejection fraction.

During a mean follow‐up of 3.6 ± 1.7 years, a total of 51 MACE (42 all‐cause death, 4 ACS requiring coronary revascularization, and 5 stroke) were observed in the entire group. Among the patients who resulted in death, 18 had cardiovascular or cerebrovascular death and the remaining had noncardiovascular or noncerebrovascular death. For the normal ABI group, all‐cause death, ACS requiring coronary revascularization, and stroke were observed in 22, 3, and 5 patients, respectively.

### 3.2 Prognostic value of biomarkers and non‐invasive examinations

In the entire group, univariate Cox hazard analysis showed that age >75 years (HR, 2.13; *P* = .012), abnormal ABI (HR, 2.42; *P* = .002), LVEF <50% (HR, 3.27; *P* = .002), upper tertile of hs‐cTnT (HR, 3.12; *P* < .001), upper tertile of hs‐CRP (HR, 1.99; *P* = .016), and upper tertile of BNP (HR, 1.90; *P* = .024) were the statistically significant predictors of MACE. Borderline significance was observed in the upper tertile of baPWV (HR, 1.68; *P* = .068). However, other variables were not associated with MACE. In multivariable model, age >75 years (HR, 2.15; *P* = .017), abnormal ABI (HR, 2.01; *P* = .020), LVEF <50% (HR, 3.33; *P* < .001), upper tertile of hs‐cTnT (HR, 2.77; *P* = .022), and upper tertile of hs‐CRP (HR, 1.96; *P* = .022) remained as the independent predictors of MACE (Table 2).

**Table 2:** Cox hazard analysis to predict MACE in the entire group (n = 104)

	Univariate analysis			Multivariable analysis		
	HR	95% CI	*P*‐value	HR	95% CI	*P*‐value
		Lower‐Upper			Lower‐Upper	
Age >75 years	2.13	1.18‐3.84	.012	2.15	1.15‐4.03	.017
PWV upper tertile	1.68	0.96‐2.94	.068	1.19	0.64‐2.20	.575
Abnormal ABI	2.42	1.37‐4.25	.002	2.01	1.11‐3.64	.020
LVEF <50%	3.27	1.80‐5.95	<.001	3.33	1.72‐6.45	<.001
BNP upper tertile	1.90	1.09‐3.32	.024	1.45	0.81‐2.59	.206
hs‐TnT upper tertile	3.12	1.79‐5.44	<.001	2.77	1.55‐4.94	.001
hs‐CRP upper tertile	1.99	1.13‐3.48	.016	1.96	1.10‐3.50	.022

*Note*: The outcome measures of baPWV and biomarkers including BNP, hs‐cTnT, and hs‐CRP were divided into tertiles of the entire group (n = 104).

Abbreviations: ABI, ankle‐brachial index; ba‐PWV, brachial‐ankle‐pulse wave velocity; BNP, brain‐natriuretic protein; CI, confidence interval; HR, hazard ratio; hs‐CRP, high‐sensitive C‐reactive protein; hs‐cTnT, high‐sensitive cardiac troponin T; hs‐TnT, high‐sensitive troponin T; LVEF, left ventricular ejection fraction; MACE, major adverse cardiovascular event; PWV, pulse wave velocity.

Similarly, univariate and multivariable Cox hazard analysis were performed to investigate the prognostic value of baPWV in the normal ABI group (Table 3). In univariate analysis, statistically significant predictors of MACE were age >75 years (HR, 3.33; *P* = .002), LVEF <50% (HR, 3.27; *P* = .002), upper tertile of hs‐cTnT (HR, 2.50; *P* = .013), upper tertile of BNP (HR, 2.07; *P* = .048), and upper tertile of baPWV (HR, 2.20; *P* = .031). Multivariable Cox hazard model demonstrated that the independent predictors of MACE were age >75 years (HR, 2.99; *P* = .010), LVEF <50% (HR, 3.05; *P* = .010), and upper tertile of hs‐cTnT (HR, 2.45; *P* = .029), whereas upper tertile of ba‐PWV was not (HR, 1.54; *P* = .293).

**Table 3:** Cox hazard analysis to predict MACE in the normal ABI group (n = 73)

	Univariate analysis			Multivariable analysis		
	HR	95% CI	*P*‐value	HR	95% CI	*P*‐value
		Lower‐Upper			Lower‐Upper	
Age >75 years	3.33	1.53‐7.27	.002	2.99	1.29‐6.90	.010
PWV upper tertile	2.20	1.07‐4.53	.031	1.54	0.68‐3.45	.293
LVEF <50%	3.27	1.52‐7.01	.002	3.05	1.30‐7.18	.010
BNP upper tertile	2.07	1.01‐4.28	.048	1.33	0.56‐3.15	.517
hs‐TnT upper tertile	2.50	1.21‐5.17	.013	2.45	1.09‐5.49	.029

*Note:* The outcome measures of baPWV and biomarkers were divided into tertiles of the normal ABI group (ABI between ≥0.9 and ≤1.4; n = 73).

Abbreviations: ABI, ankle‐brachial index; ba‐PWV, brachial‐ankle‐pulse wave velocity; BNP, brain‐natriuretic protein; CI, confidence interval; HR, hazard ratio; hs‐TnT, high‐sensitive troponin T; LVEF, left ventricular ejection fraction; MACE, major adverse cardiovascular event; PWV, pulse wave velocity.

**Figure 1:**
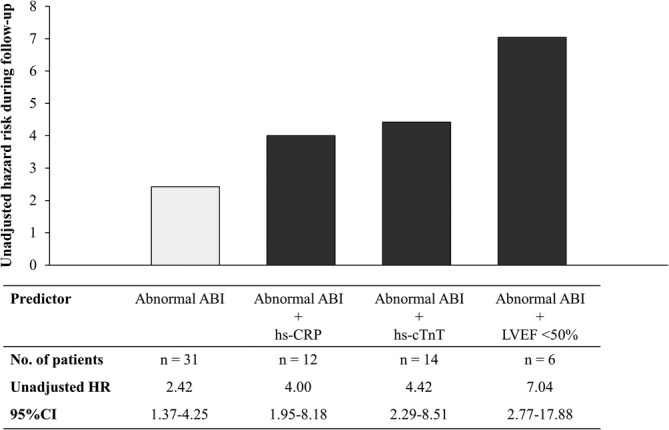
The figure shows hazard ratio and 95% confidence interval of abnormal ABI and combination of independent predictors of MACE obtained by univariate Cox regression analysis for the entire group (n = 104). Combination of predictors in addition to abnormal ABI (HR, 2.42; 95% CI 1.37‐4.25; *P* = .002) showed increased hazard ratio for abnormal ABI + upper tertile of hs‐CRP (HR, 4.00; 95% CI 1.95‐8.18; *P* < .001), abnormal ABI + upper tertile of hs‐cTnT (HR, 4.42; 95% CI 2.29‐8.51; *P* < .001), and abnormal ABI + LVEF <50% (HR, 7.04; 95% CI 2.77‐17.88; *P* < .001). The cutoff values for the upper tertile of hs‐TnT and hs‐CRP were 0.081 and 0.17 mg/dL, respectively. ABI, ankle‐brachial index; CI, confidence interval; CRP, C‐reactive protein; cTnT, cardiac troponin T; HR, hazard ratio; hs, high‐sensitive; LVEF, left ventricular ejection fraction; MACE, major adverse cardiovascular event

**Figure 2:**
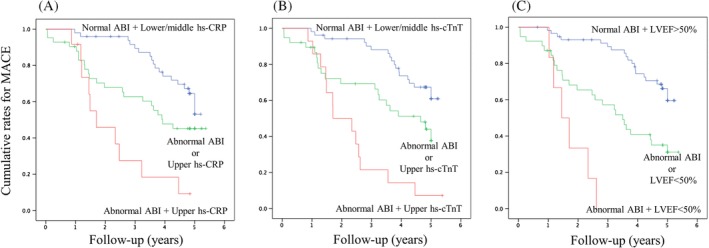
Kaplan–Meier curves analysis to predict MACE in the entire group stratified by the presence or absence of predictors. A, Presence or absence of predictors, including abnormal ABI and the upper tertile of hs‐CRP (Log‐rank test, *P* < .001). B, Presence or absence of predictors including abnormal ABI and the upper tertile of hs‐cTnT (Log‐rank test, *P* < .001). C, Presence or absence of predictors, including abnormal ABI and LVEF <50% (Log‐rank test, *P* < .001). The cutoff values for the upper tertile of hs‐TnT and hs‐CRP were 0.081 and 0.17 mg/dL, respectively. ABI, ankle‐brachial index; CI, confidence interval; CRP, C‐reactive protein; cTnT, cardiac troponin T; HR, hazard ratio; hs, high‐sensitive; LVEF, left ventricular ejection fraction; MACE, major adverse cardiovascular event

The combination of abnormal ABI with other independent predictors of MACE exhibits higher HR compared to the abnormal ABI alone (Figure 1). Kaplan‐Meier analyses demonstrated a graded risk of MACE when classified according to the presence or absence of predictors in addition to abnormal ABI (Figure 2).

## 4 DISCUSSION

This study demonstrated that abnormal ABI was a strong predictor of MACE independent of biomarkers in ESKD patients, whereas ba‐PWV was not. Furthermore, the combination of abnormal ABI with biomarkers and LVEF showed a greater hazard risk by complementing the ABI examination for the prediction of MACE in ESKD patients treated with HD.

Previous clinical studies have demonstrated that abnormal ABI and biomarkers are robust predictors of mortality and cardiovascular outcomes in ESKD patients.^12^, ^15^, ^16^, ^17^, ^18^, ^19^, ^20^ Tanaka et al, demonstrated that the presence of abnormal ABI is a strong predictor of PAD outcomes, all‐cause mortality, and cardiovascular events in ESKD patients.^12^ Several studies have reported that multiple‐biomarker approach enabled to provide better risk stratification of ESKD patients.^15^, ^16^, ^17^, ^18^, ^19^, ^20^ In consistent with the previous studies, we observed that upper tertile of hs‐TnT and hs‐CRP are independently associated with MACE for the overall population of ESKD. However, the prognostic value of ABI/PWV in association with biomarkers has not been fully investigated in ESKD patients.

In the present study, we found that abnormal ABI was a strong predictor of MACE independent of biomarkers, whereas baPWV was not an independent predictor for the entire group and also for the patients with normal ABI. baPWV has been reported to be correlated with the direct measure of aortic PWV and carotid‐femoral PWV, which has been shown to be a strong predictor of mortality in ESKD patients.^10^, ^23^ However, because of technical issues such as measurement protocols and reproducibility,^22^ the use of baPWV for the accurate risk prediction of ESKD patients is still discussed.^14^ Ho‐Ming, et al showed that the ba‐PWV measurements were influenced by blood volume changes after HD sessions, whereas ABI was not.^11^ These observations support the use of ABI for the risk stratification of ESKD patients treated with HD.

Furthermore, we found that the combination of upper tertile of hs‐cTnT or hs‐CRP in addition to abnormal ABI showed higher risk compared to abnormal ABI alone. A possible explanation is that elevations of these biomarkers represent the target organ damage and systemic inflammation, which is difficult to be evaluated by ABI alone. Abnormal ABI has been shown to be well correlated with systemic disease burden of atherosclerosis, which is a strong predictor of MACE.^6^, ^8^ On the other hand, accumulating evidence demonstrated that an elevation of cTn is specific to myocardial damage^24^, ^25^, ^26^ and hs‐CRP reflects systematic atherosclerotic burden and activity even in ESKD patients.^1^, ^17^, ^27^ As multiple organ disorder causes markedly higher systemic adverse events,^2^, ^3^, ^4^ these observations suggest that biomarker approach complements the risk assessment that cannot be identified by ABI alone, and their combination could provide better risk stratification of ESKD patients treated with HD.

In addition, we observed that decreased LVEF remained as an independent predictor of MACE in both the entire group and the normal ABI group. Decreased LVEF was closely associated with cTn elevation and both were important predictors of mortality and cardiovascular prognosis in ESKD patients.^18^, ^24^, ^26^ In a clinical study using positron emission tomography, Shah, et al. demonstrated that ESKD patients with impaired global coronary flow reserve had a lower LVEF compared with those without impaired coronary flow reserve.^28^ These observations may partly explain our finding that decreased LVEF that potentially coexist with impaired microvascular dysfunction was a robust predictor of MACE in this population. Moreover, we observed that the highest risk of MACE was among the patients with abnormal ABI and decreased LVEF. It is plausible that the presence of PAD and decreased LVEF are associated with limited capacity of exercise tolerance and impaired cardiopulmonary function,^29^, ^30^, ^31^ which is a potential mechanism for increased risk of MACE.

The findings of this study suggest that the use of ABI rather than baPWV may provide prognostic information independent of other robust predictors such as decreased LVEF and biomarkers in this population. Considering its noninvasive nature and its common use in the clinical practice of ESKD patients, ABI and biomarker approach could be of importance to the management and the risk prediction of ESKD patients. Future large population studies are needed to determine if the combination of these independent predictors provides better risk prediction of MACE in ESKD patients treated with HD.

## 5 STUDY LIMITATIONS

Our study has several limitations. First, this study included relatively small number of patients with and without normal ABI. Our findings regarding the predictors of MACE should be interpreted with caution, although baPWV did not remain as an independent predictor of MACE even in the normal ABI group. Second, we employed baPWV as a marker of arterial stiffness because of its common use in clinical practice. Different markers such as aortic stiffness and arterial stiffness gradient to assess arterial stiffness may contribute to the prediction of MACE in association with biomarkers and other noninvasive tests.^7^ Finally, we evaluated the markers at a single time point of the study enrollment. Arterial stiffness is associated with organ damage which cannot be entirely explained by the established cardiovascular risk factors.^32^ Several studies have suggested the potential benefit to assess changes in arterial stiffness as the treatment response. Future studies are needed to investigate the impact of targeting changes in arterial stiffness after optimized therapy in predicting MACE in ESKD patients.

## 6 CONCLUSIONS

This study demonstrated that abnormal ABI was a robust predictor of MACE independent of biomarkers and decreased LVEF, whereas ba‐PWV was not. The combination of the presence or absence of these predictors could provide better risk stratification compared with abnormal ABI alone in ESKD patients treated with HD.

## CONFLICT OF INTEREST

The authors declare no potential conflict of interests
